# Transcriptomic analysis of diplomonad parasites reveals a trans-spliced intron in a helicase gene in *Giardia*

**DOI:** 10.7717/peerj.2861

**Published:** 2017-01-05

**Authors:** Scott William Roy

**Affiliations:** Department of Biology, San Francisco State University, San Francisco, CA, United States

**Keywords:** Genome complexity, Trans-splicing, Protist molecular biology

## Abstract

**Background:**

The mechanisms by which DNA sequences are expressed is the central preoccupation of molecular genetics. Recently, ourselves and others reported that in the diplomonad protist *Giardia lamblia*, the coding regions of several mRNAs are produced by ligation of independent RNA species expressed from distinct genomic loci. Such trans-splicing of introns was found to affect nearly as many genes in this organism as does classical cis-splicing of introns. These findings raised questions about the incidence of intron trans-splicing both across the *G. lamblia*transcriptome and across diplomonad diversity in general, however a dearth of transcriptomic data at the time prohibited systematic study of these questions.

**Methods:**

I leverage newly available transcriptomic data from *G. lamblia* and the related diplomonad *Spironucleus salmonicida*to search for trans-spliced introns. My computational pipeline recovers all four previously reported trans-spliced introns in *G. lamblia*, suggesting good sensitivity.

**Results:**

Scrutiny of thousands of potential cases revealed only a single additional trans-spliced intron in *G. lamblia*, in the p68 helicase gene, and no cases in *S. salmonicida*. The p68 intron differs from the previously reported trans-spliced introns in its high degree of streamlining: the core features of *G. lamblia* trans-spliced introns are closely packed together, revealing striking economy in the implementation of a seemingly inherently uneconomical molecular mechanism.

**Discussion:**

These results serve to circumscribe the role of trans-splicing in diplomonads both in terms of the number of genes effected and taxonomically. Future work should focus on the molecular mechanisms, evolutionary origins and phenotypic implications of this intriguing phenomenon.

## Introduction

Splicing of nuclear RNA transcripts by the spliceosomal machinery is a ubiquitous feature of the expression of nuclear genes in eukaryotes (Roy & Irimia, 2014; Nixon et al., 2002; Vanácová et al., 2005; although see Lane et al., 2007; Akiyoshi et al., 2009). Splicing within protein-coding sequences nearly always joins two protein-coding regions of a single RNA transcribed from a single locus: intron *cis-*splicing (Chow et al., 1977). Alternatively, protein-coding regions from multiple RNAs transcribed from different loci can be joined: intron *trans*-splicing (Li et al., 2009; Takahara et al., 2000; Dorn, Reuter & Loewendorf, 2001; Robertson et al., 2007; Fang et al., 2012) (This process should be distinguished from spliced leader trans-splicing, in which a short non-coding RNA molecule is added to various mRNAs outside of the coding region, essentially donating 5′ UTR sequence (Lasda & Blumenthal, 2011)). *Trans*-splicing of introns is generally very rare: for instance, among the hundreds of thousands of known splicing events in humans, there are fewer than 10 confirmed cases of genic trans-splicing (Wu et al., 2014). Recently, the first case in which a substantial fraction of introns in an organism are *trans*-spliced was reported. In the genome of the diplomonad intestinal parasite *G. lamblia*, systematic studies have revealed only six *cis*-spliced introns to date (Nixon et al., 2002; Russell et al., 2005; Morrison et al., 2007; Roy et al., 2012; Franzén et al., 2013); intriguingly small-scale studies revealed four cases of genic trans-splicing, including two in a single gene (Nageshan et al., 2011; Kamikawa et al., 2011; Roy et al., 2012; Hudson et al., 2015). These cases showed distinctive sequence features—most notably extended basepairing potential between the pairs of trans-spliced transcripts.

These studies raised two clear questions. First, given the fact that these cases were found largely serendipitously, with a single gene containing two separate trans-spliced introns, is genic trans-splicing in *G. lamblia* much more widespread? Second, what is the evolutionary history of trans-splicing in *G. lamblia* and other diplomonads? However, the lack of availability of large amounts of mRNA sequence data at that time prohibited systematic study of these questions. Recently, Franzén et al. (2013) reported a transcriptome analysis of three different strains of *G. lamblia* and Xu et al. (2014) reported the genome and transcriptome of the distantly-related diplomonad parasite *Spironucleus salmonicida.* Here, I report the first transcriptome-wide studies of intron *trans*-splicing in *G. lamblia* isolates and *S. salmonicida*

## Methods

Full genome sequences and Illumina RNA-seq data were downloaded for three strains of *G. lamblia* (GEO accession GSE36490, from Franzén et al., 2013) and for *S. salmonicida* (SRA accession SRR948595, from Xu et al., 2014). Bowtie (Langmead et al., 2009) was used with default parameters to exclude read pairs that mapped in expected orientation to the genome (with a maximum insert size 1,000 nucleotides) and as well as individual reads that mapped to the genome. I then mapped the non-mapping reads to the genome using blat (Kent, 2002) and identified reads for which (i) parts of the read mapped in exactly two places; (ii) both the 5′ and 3′ termini of the read mapped (that is, the mapping started within 5 nucleotides of the end of the read); and (iii) the junction between the two mappings was relatively precise –a single unambiguous junction with five or fewer nucleotides of overlap (i.e., in cases of similarity between the genomic sequences at the boundaries of the junction) or of gap (i.e., nucleotides near the junction that are not represented in either genomic locus). Junctions supported by at least two reads that suggested trans-splicing (either >5 kb apart on the same contig or on different contigs) were then collected.

Each potential case of trans-splicing was assigned a 5′ and 3′ score based on adherence to splice boundaries of previously reported introns. Scores were calculated using a standard PWM approach as follows: (i) 5′ and 3′ splice sites were compiled for all known *cis*- and *trans*-spliced introns for both species (seven and 14 intronic nucleotides respectively for *G. lamblia*; 11 and 21 intronic nucleotides respectively for the longer conserved consensus sequences of *S. salmonicida*); (ii) for each position within the boundary, each of the four nucleotides was assigned a score equal to the frequency of the nucleotide at that position in known introns, plus 0.05 (added to account for the possibility that newly found introns could use nucleotides not observed among the small sets of known introns); (iii) the raw score for each boundary for each potential trans-splicing case was calculated as the log of the product of the scores across sites; (iv) the final score was calculated as the maximum possible score minus the raw score (thus the maximum possible final score is zero). Scores were calculated for each position within five nucleotides downstream and upstream of the apparent junction, and the maximum among these scores was used as the score for the potential trans-splicing case. In addition, for both species, each potential case of trans-splicing was analyzed by eye. To determine evidence for trans-splicing in the various datasets, 12 RNA-seq datasets from Ansell et al. (2015) were downloaded from SRA (Accession PRJNA298647). The first 100 nucleotides of each read for the Franzen et al. and Ansell et al. datasets were mapped against the spliced and unspliced forms of each trans-spliced intron using Bowtie with default parameters, with reads that mapped to only the spliced form being taken as evidence for splicing. Putative *S. salmonicida* orthologs of trans-spliced *G. lamblia* genes were identified by reciprocal BLASTP searches.

## Results and Discussion

### Transcriptomic analysis of trans-splicing in diplomonad parasites

I downloaded 11 Illumina RNA-seq datasets from previous transcriptomic analyses, 10 for *G. lamblia* parasites from Franzén et al. (2013) and one of *S. salmonicida* from Xu et al. (2014). For each species, I used bowtie and blat to identify Illumina reads that contained sequence from multiple genomic loci and which are suggestive of *trans*-splicing (see ‘Methods’). This procedure identified some 495,066 potential boundaries in *G. lamblia* and 231,769 in *S. salmonicida* For both species, the vast majority of these cases were either supported by only a single read (400,460 and 212,801 respectively), had extended similarities at the 5′ and 3′ boundaries suggesting reverse transcriptase artifacts produced during library formation (‘RTfacts’; Roy & Irimia, 2008) (388,835 and 159,836 cases), and/or did not represent a clear splice junction (with >5 nucleotides in the middle of the read that did not map to either locus (35,740 and 8,307 cases). Filtering of these dubious cases left 2,272 potential boundaries in *G. lamblia* and 5,454 in *S. salmonicida*.

**Figure 1:**
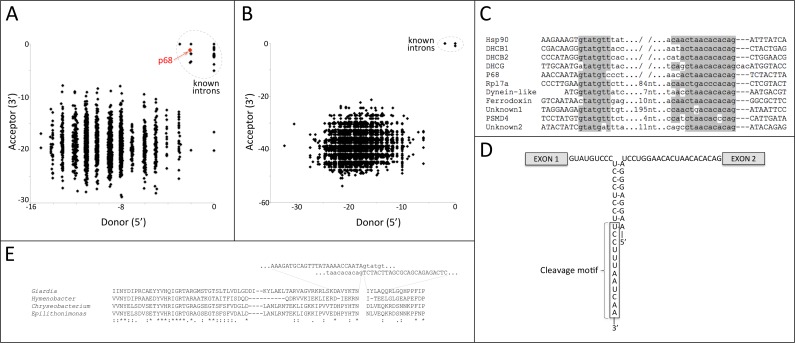
Transcriptome-wide search identifies a trans-spliced intron in p68 helicase. (A) Normalized PWM scores for similarity to known donor and acceptor intron splice boundaries for 2272 potential *G. lamblia* trans-splicing events supported by at least two reads and for known *cis-* and *trans-*spliced introns. Only one potential trans-splicing events, in the p68 helicase, groups with known introns. Boundaries are scored relative to the maximum possible score (equating to zero). (B) Normalized PWM scores for similarity to known donor and acceptor intron splice boundaries for 5454 potential *S. salmonicida* trans-splicing events supported by at least two reads and for known *cis*-spliced introns. No potential trans-splicing events group with known introns. (C) Comparison of splice boundaries for newly-discovered p68 trans-spliced intron with known trans- and cis-spliced introns, for *G. lamblia* isolate GS. (D) Trans-spliced intron sequences for newly-discovered p68 intron exhibits basepairing potential between intronic regions of 5′ and 3′ pre-mRNA transcripts and conserved cleavage motif reported by Hudson et al. (2012). (E) Protein sequence alignment between the protein encoded by trans-spliced *G. lamblia* p68 gene and highest-scoring BLAST hits in Genbank (*Chryseobacterium caeni,* Accession WP_027384510.1, *Hymenobacter sp. AT01-02*, Accession WP_052694982.1, and *Epilithonimonas tenax,* Accession WP_028122041.1).

All of these cases were analyzed by eye for presence of sequences corresponding to extended 5′ or 3′ splicing signals particular to the species. In *G. lamblia,* this analysis yielded five clear cases in *G. lamblia* and no “borderline” cases. That is, each of the five cases had an extended 5′ splicing signal (consensus GTATGTT), an extended 3′ splicing signal (CT[AG]ACACACAG), complementarity between the pairs of apparently trans-spliced loci, and presence of the *G. lamblia* 3′ cleavage motif (consensus sequence TCCTTTACTCAA); no other cases showed any of these features. To confirm this manual analysis, all potential boundaries were also analyzed for adherence of splicing motifs to those of all known *cis*- and *trans*-spliced introns (10 total in *G. lamblia*, 4 total in *S. salmonicida*), using a position weight matrix (PWM) approach (Fig. 1A). These automated analyses confirmed the findings of only a single new case that exhibited canonical splicing boundaries. A similar combination of manual and automated PWM analysis in *S. salmonicida* did not yield any strong trans-splicing candidates (Fig. 1B). In addition, no instances of trans-splicing were found in the putative *S. salmonicida* orthologs of the five trans-spliced *G. lamblia* genes (four previously reported and the one novel case reported here (see below)).

### A trans-spliced intron in a p68 helicase gene

I next focused on the five identified trans-splicing candidates in *G. lamblia.* Mapping of RNA-seq data from Franzén et al. (2013) and from a second study that became available during the course of this project (Ansell et al., 2015) revealed direct RNA support for all five of these trans-splicing events across various *G. lamblia* isolates and conditions, with between 388 and 55,177 total reads supporting the five cases (Table 1). Four of these cases corresponded to all four previously reported trans-spliced introns. The fifth case represents a previously unreported case of trans-splicing, falling in a putative p68 RNA-dependent helicase gene. Figure 1C shows a comparison between splice boundaries for the newly discovered trans-spliced intron and all previously reported introns.

**Table 1:** Number of reads supporting trans-splicing of five trans-spliced *G. lamblia* introns from mixed stage or synchronized stage trophozoites from 22 Illumina RNA-seq datasets. 48hr/60hr/96hr-Troph indicate hours after beginning of the trophozoite stage (for details, see Ansell et al. (2015)). DHCB1/2, first/second intron of dynein heavy chain beta; DHCG, dynein heavy chain gamma.

Dataset	Isolate	Stage	DHCB1	DHCB2	DHCG	HSP90	P68
SRR455165	WB	Trophozoit	72	105	29	871	13
SRR455166	WB	Trophozoit	83	94	31	891	17
SRR455169	WB	Trophozoit	64	274	18	2,190	13
SRR455170	WB	Trophozoit	59	293	12	2,271	12
SRR455171	WB	Trophozoit	39	294	6	3,849	22
SRR455172	WB	Trophozoit	33	281	4	3,843	10
SRR455167	P15	Trophozoit	40	97	15	1,164	27
SRR455168	P15	Trophozoit	51	74	15	1,103	26
SRR455173	GS	Trophozoit	7	184	37	2,726	1
SRR455174	GS	Trophozoit	18	176	28	2,858	1
SRR2642193	WB1B	48hr-Troph	1	179	2	2,498	10
SRR2642194	WB1B	48hr-Troph	0	102	0	614	5
SRR2642197	WB1B	48hr-Troph	8	462	1	2,835	20
SRR2642198	WB1B	48hr-Troph	134	1,122	36	5,444	18
SRR2642199	WB1B	60hr-Troph	13	966	1	2,675	37
SRR2642200	WB1B	60hr-Troph	111	1,427	33	2,697	7
SRR2642201	WB1B	60hr-Troph	3	117	0	793	3
SRR2642202	WB1B	60hr-Troph	210	1,560	37	1,998	6
SRR2642195	WB1B	96hr-Troph	6	182	1	487	21
SRR2642196	WB1B	96hr-Troph	1,114	3,041	76	5,590	42
SRR2642204	WB1B	96hr-Troph	767	3,276	66	4,491	40
SRR2642205	WB1B	96hr-Troph	939	3,418	76	3,289	37
**Total**			**3,772**	**17,724**	**524**	**55,177**	**388**

This new trans-spliced intron exhibits the characteristic traits of the four previously-reported trans-spliced introns: (i) extended 5′ and 3′ splice sites (GTATGT and ACTAACACAG, respectively; Fig. 1D); (ii) extended basepairing between the intronic regions of the two pre-mRNA transcripts (Fig. 1D); and (iii) the recently-discovered *G. lamblia* cleavage motif (with consensus TCCTTTACTCAA; Fig. 1D; Hudson et al., 2012). A BLASTX search of the mature trans-spliced transcript against Genbank revealed homology to p68 helicase (Fig. 1E). Whereas previous trans-spliced introns were found to lie at the boundaries of genic regions encoding domains (Roy et al., 2012), the p68 trans-spliced intron falls outside of conserved regions (Fig. 1E), which prohibited me from determining the relationship of the splicing position to encoded protein domain structure.

This newly discovered intron exhibits a more compact structure than previously reported trans-spliced introns, with a short stretch of perfect Watson-Crick basepairing directly followed by (indeed, overlapping) the cleavage motif (Fig. 1D). For comparison, the cleavage motif in the p68 intron lies only 17 nucleotides downstream of the 5′ splice site, compared to 34–93 nucleotides in the four previously-described *trans*-spliced introns.

### The extent of intron trans-splicing in time and space

The finding that our transcriptomic pipeline was able to identify all four previously reported cases of *G. lamblia* trans-splicing suggests that the pipeline does not have a very high false negative rate. As such, that the pipeline identified only a single additional case of trans-splicing suggests that the breadth of trans-splicing within the *G. lamblia* transcriptome may be limited. Similarly, that the pipeline did not identify promising trans-splicing candidates in *S. salmonicida* further suggests that the phylogenetic breadth of trans-splicing within diplomonads may similarly be limited (consistent with the findings of Xu et al. (2014)). Future work should focus on better understanding the diversity and origins of trans-splicing within relatives of *G. lamblia*.

## Conclusions

These results add to the set of known trans-spliced introns in *G. lamblia* while at the same time circumscribing the likely transcriptome-wide importance of trans-splicing in this organism. The structural simplicity of the reported p68 helicase intron reveals a degree of economy in implementing the seemingly uneconomical inefficient molecular mechanism of trans-splicing. These cases together represent a further embellishment on the core mechanisms of gene expression. As with previously described embellishments—intron splicing, alternative splicing and promoter usage, spliced leader trans-splicing, ribosomal readthrough and frameshifting, etc.—attention now turns to understanding the mechanisms, evolutionary origins and potential phenotypic implications of these intriguing trans-spliced introns.

## Additional Information and Declarations

### Data Availability

The following information was supplied regarding data availability: Full genome sequences and Illumina RNA–seq data were downloaded for three strains of *G. lamblia* (GEO accession GSE36490, from Franzén et al., 2013) and for *S. salmonicida* (SRA accession SRR948595, from Xu et al., 2014).
